# CEACAM6 Promotes Gastric Cancer Invasion and Metastasis by Inducing Epithelial-Mesenchymal Transition via PI3K/AKT Signaling Pathway

**DOI:** 10.1371/journal.pone.0112908

**Published:** 2014-11-14

**Authors:** Mingde Zang, Baogui Zhang, Yunqiang Zhang, Jianfang Li, Liping Su, Zhenggang Zhu, Qinlong Gu, Bingya Liu, Min Yan

**Affiliations:** Department of Surgery, Shanghai Key Laboratory of Gastric Neoplasms, Shanghai Institute of Digestive Surgery, Ruijin Hospital, Shanghai Jiao Tong University School of Medicine, Shanghai, People’s Republic of China; University of Hong Kong, Hong Kong

## Abstract

Overexpressed CEACAM6 in tumor tissues plays important roles in invasion, metastasis and anoikis resistance in a variety of human cancers. We recently reported that CEACAM6 expression is upregulated in Gastric cancer (GC) tissues and promoted GC metastasis. Here, we report that CEACAM6 promotes peritoneal metastases *in* *vivo* and is negatively correlated with E-cadherin expression in GC tissues. Overexpressed CEACAM6 induced epithelial-mesenchymal transition (EMT) in GC, as measured by increases in the EMT markers N-cadherin, Vimentin and Slug while E-cadherin expression was decreased in CEACAM6-overexpressing GC cells; opposing results were observed in CEACAM6-silenced cells. Furthermore, E-cadherin expression was negatively correlated with depth of tumor invasion, lymph node metastasis and TNM stage in GC tissues. Additionally, CEACAM6 elevated matrix metalloproteinase-9 (MMP-9) activity in GC, and anti-MMP-9 antibody could reverse the increasing invasion and migration induced by CEACAM6. CEACAM6 also increased the levels of phosphorylated AKT, which is involved in the progression of a variety of human tumors. We further observed that LY294002, a PI3K inhibitor, could reverse CEACAM6-induced EMT via mesenchymal-epithelial transition. These findings suggest that CEACAM6 enhances invasion and metastasis in GC by promoting EMT via the PI3K/AKT signaling pathway.

## Introduction

GC is one of the most common malignant tumors and a major health issue worldwide, particularly in East Asian countries such as Japan, Korea, China, where it is the second cause of cancer-related death [1]–[3]. Carcinoembryonic antigen-related cell adhesion molecule 6 (CEACAM6) is a glycosylphosphatidylinositol (GPI)-linked immunoglobulin superfamily member that is overexpressed in a variety of human cancers, especially gastrointestinal cancers [4], and functions as an intercellular adhesion molecule [4]–[6]. Although CEACAM6 is a GPI-anchored cell surface glycoprotein it lacks a transmembrane or intracellular domain, but is able to influence intracellular signaling events and plays an important role in gastrointestinal cancer progression [4], [6]–[8]. GPI-anchored molecules are often co-localized to small membrane microdomains in the plasma membrane [9], which can activate downstream signaling cascades such as the integrin signaling pathway [10]. CEACAM6 acts as an oncogene in tumors and promotes cancer invasion, metastasis, anoikis resistance and chemoresistance, and inhibits differentiation [7], [8], [11]. We recently reported that CEACAM6 expression is upregulated and associated with lymph node metastasis in GC tissues [12]. However, the mechanisms through which CEACAM6 influences intracellular signal transduction in GC remain to be determined.

Epithelial-mesenchymal transition (EMT) is not only a physiological process during embryonic development or tissue regeneration, but also a pathological element in cancer progression, involving tumor metastasis, apoptosis and senescence resistance [13]–[15]. EMT always indicates a poor clinical prognosis in human cancers. A number of mechanisms relevant to EMT initiation have been documented, including the TGF-β, IL-6, PI3K/AKT, RAF/MAPK, and SRC pathways [15]–[18]. In our previous study, we observed CEACAM6-induced SRC activation in GC cells [12], and observed increased numbers of spindle-shaped CEACAM6-overexpressing cells compared with control cells. Hence, we were very interested in determining the relationship between CEACAM6 and EMT in GC cells. Although negative correlation between CEACAM6 and EMT has been recorded in pancreatic carcinomas [19], the mechanisms by which CEACAM6 regulates EMT are poorly understood.

In this study, we further examined the effects and potential pathways of CEACAM6 in GC invasion and metastasis, and investigated its correlation with EMT.

## Materials and Methods

### Ethics Statement

Written informed consent in the study has been obtained from all participants. The study protocol was approved by the ethics committee of Ruijin Hospital, Shanghai Jiao Tong University School of Medicine. Animal procedures were carried out according to a protocol approved by the Institutional Animal Care and Use Committee (IACUC) at Shanghai Jiao Tong University, Shanghai, China.

### Cell lines and tissue specimens

The human GC cell lines SGC-7901, MKN-45 and MKN-28 were purchased from Shanghai Institutes for Biological Sciences, Chinese Academy of Sciences. Cells were cultured at 37°C in 5% CO_2_ and saturation humidity in RPMI-1640 medium containing 10% fetal bovine serum.

Gastric tumor and adjacent non-tumorous tissue were obtained from 93 patients with GC who underwent curative surgery at Shanghai Jiaotong University School of Medicine Affiliated Ruijin Hospital from 2010 to 2013. The patients consisted of 69 men and 24 women with a mean age of 62.1 years (range, 30–85 years). None of the patients had received radiotherapy or chemotherapy prior to surgery. Clinicopathological data were collected and pathological tumor staging was determined according to the UICC TNM classification. Histological typing was performed by at least two expert pathologists working independently in a double-blinded fashion. This study was approved by the Ethics Committee of Shanghai Ruijin Hospital, and all patients were fully informed of the experimental procedures.

### Vector construction and transfection

Full-length CEACAM6 cDNA was obtained by RT-PCR from total RNA extracted from GC samples. The primer sequences were 5′-CCGGAATTCCCATGGGACCCCCCTCAGCCC-3′ (forward) and 5′-TCCCCCGGGGCTATATCAGAGCCACCCTGG-3′ (reverse). We assembled a pIRES2-eGFP-CEACAM6 construct by inserting CEACAM6 cDNA into pIRES2-eGFP vector. We next transfected pIRES2-eGFP-CEACAM6 or pIRES2-eGFP vector into SGC-7901 and MKN-45 cells using Lipofectamine 2000 (Invitrogen, Carlsbad, USA) in accordance with the manufacturer’s protocol. Stable clones were selected by continuous treatment with G418 (1.2 mg/ml; Gibco, New York, USA).

### Lentiviral vector construction

Based on human CEACAM6 gene data, a pair of oligonucleotide sequences and negative control sequences were designed and synthesized by Shanghai Novobio Scientific Co., Ltd. shRNA sequences were as follows: CEACAM6, 5′-CACCGCCGGACAGTTCCATGTATACGAATATACATGGAACTGTCCGG-3′ (forward), and 5′-AAAACCGGACAGTTCCATGTATATTCGTATACATGGAACTGTCCGGC-3′ (reverse); control, 5′-CACCGCTACACAAATCAGCGATTTCGAAAAATCGCTGATTTGTGTAG-3′ (forward), and 5′-AAAACTACACAAATCAGCGATTTTTCGAAATCGCTGATTTGTGTAGC-3′ (reverse). CEACAM6 shRNA was subcloned into pL/shRNA/F lentiviral vector to obtain a pL/shRNA/shR-CEACAM6 construct. Then pL/shRNA/shR-CEACAM6 or control lentiviral vectors were transfected into MKN-28 GC cells. Stably transfected cells were selected by treatment with 5 µg/ml blasticidin and were used for identification and further research.

### Western blotting

Cells were harvested and lysed using RIPA buffer (Solarbio, Beijing, China) containing 1% PMSF protease inhibitors. A BCA assay kit (Pierce, Rockford, USA) was used to measure the total protein concentration. Equivalent amounts of protein were separated by 10% SDS-PAGE, and the resolved proteins transferred to PVDF membranes. The membranes were blocked with 5% skim milk for 2 h and then incubated with primary antibodies overnight at 4°C. Primary antibodies were as follows: CEACAM6 (1∶500; Abcam, USA), E-cadherin (1∶500; Cell Signaling Technology (CST), USA), N-cadherin (1∶500; CST, USA), Vimentin (1∶1000; CST, USA), Slug (1∶500; CST, USA), p-AKT (Ser473) (1∶1000; CST, USA), total AKT (1∶1000; CST, USA) and GAPDH (1∶10000; Abcam, USA). Membranes were then incubated with secondary antibody for 2 h at room temperature and were visualized using an enhanced chemiluminescence detection system (Amersham Bioscience, Piscataway, NJ, USA) in accordance with the manufacturer’s protocol.

### Immunofluorescent staining

Cells were cultured on coverslips for 24 h and then fixed with 4% paraformaldehyde for 15 min. Cells were washed with PBS, then permeabilized with 0.5% Triton X-100 for 20 min and blocked with 5% BSA for 30 min at room temperature. We next stained the cells with CEACAM6 antibody (1∶50; Abcam) at 37°C for 2 h, followed by incubation with fluorescent secondary antibody for 1 h at room temperature. The nuclei were stained with DAPI. Rhodamine phalloidin antibody (1∶150; Cytoskeleton) was used to visualize the cytoskeleton of GC cells. Slides were analyzed and imaged on a fluorescence microscope.

### Immunohistochemistry

Sections of 4 µm-thick were cut from paraffin-embedded tissue blocks and then deparaffinized and rehydrated. Immunohistochemical staining of sections was performed according to the DAKO protocol, using mouse anti-CEACAM6 (1∶100; Abcam) and E-cadherin (1∶100; CST) at 4°C overnight. Slides were then incubated with HRP-labeled secondary antibody and visualized by diaminobenzidine. Two pathologists who were blinded to any patient data independently evaluated and scored the sections. Immunohistochemistry stain score = positive cell score + staining intensity score. The percentage of positive cells was scored as follows: 0 (10%), 1 (10–25%), 2 (26–50%), 3 (51–75%) and 4 (>75%). Immunohistochemical staining intensity was graded as follows: 0 (no staining), 1 (weak staining), 2 (brown staining), and 3 (dark brown staining). Total scores of ≥3 were defined as positive to simplify data analysis. To analyze the correlation between CEACAM6 and E-cadherin expression, Image-Pro Plus version 6.0 (Media Cybernetics, Inc., Bethesda, MD, USA) was used to quantify the expression of CEACAM6 and E-cadherin on 20 samples.

### Gelatin zymography

To examine MMP-9 activity, zymography was performed using 10% SDS-PAGE gels containing 1 mg/ml gelatin (Sigma). Briefly, GC cells were cultured in serum-free RPMI-1640 medium for 24 h and the supernatant was then collected and centrifuged at 1000 rpm for 5 min. The gels were washed twice in renaturation buffer (2.5% Triton X-100) for 30 min each time to remove SDS after electrophoresis and then incubated at 37°C for 24 h in a reaction buffer (50 mM Tris–HCl pH 7.5, 5 mM CaCl2, 150 mM NaCl). We next stained the gels with 0.5% Coomassie brilliant blue R-250 for 2 h and destained the gels in buffer (30% methanol, 10% acetic acid). Clear transparent bands in the background of blue staining represented the gelatinase activities.

### Cell migration and invasion assays

For cell migration and invasion assays, a total of 1×10^5^ cells were suspended in serum-free RPMI-1640 with or without MMP-9 antibody (Abcam, 2 µg/200 ul) and plated in transwell chambers (8 µm for 24-well plate; Corning Costar, NY, USA) with or without Matrigel (BD Bioscience, CA, USA) according to the manufacturer’s protocols. For both assays, medium containing 10% FBS was added to the lower chamber as a chemoattractant. After 24 h culture, cells were fixed by 10% formalin and stained by 0.5% crystal violet. Finally, cells in the lower chamber were photographed and counted by inverted microscopy.

### Nude mouse xenograft model

Four-week-old male BALB/c nude mice (Institute of Zoology, China Academy of Sciences) were used to evaluate the role of CEACAM6 in peritoneal spreading *in* *vivo*. Nude mice were housed at a specific pathogen-free environment in the Animal Laboratory Unit, School of Medicine, Shanghai Jiao Tong University, China. Mice received humane care and the study protocols were carried out according to a protocol approved by the Institutional Animal Care and Use Committee (IACUC) at Shanghai Jiao Tong University, Shanghai, China. A total of 2×10^6^ SGC-7901-CEACAM6 or SGC-7901-NC cells were injected into five mice abdominal cavity of each group (randomisation). Mice were euthanized on the 30th day after injection, and the abdominal masses were imaged and photographed. All experiments were performed in accordance with the official recommendations of the Chinese animal community.

### Statistics

Student’s *t*-test was used to examine the statistical differences between the two groups. Correlations between E-cadherin expression in GC tissues and clinicopathological parameters and CEACAM6 expression were analyzed by chi-square or Fisher’s exact tests or Pearson correlation coefficient analysis. Data are shown as mean±SD. *P*<0.05 and *P*<0.01 was considered statistically significant and highly significant, respectively.

## Results

### CEACAM6 affects cell morphology and the cytoskeleton

**Figure 1:**
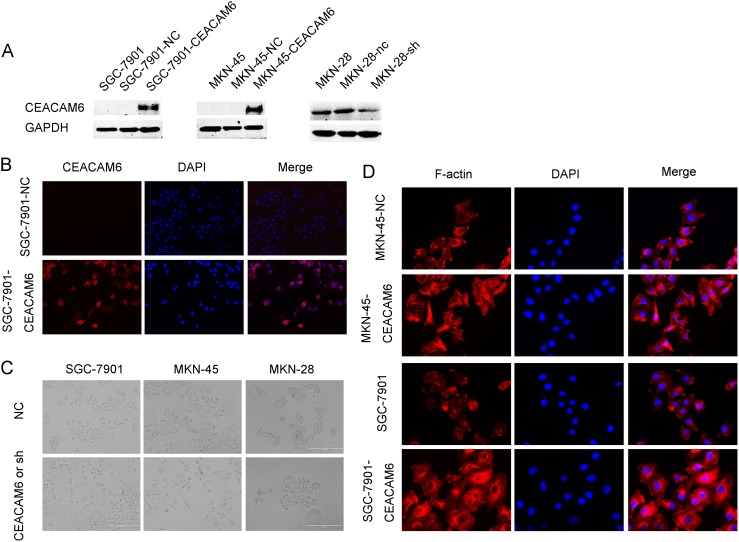
Effects of CEACAM6 on cell morphology and cytoskeleton in GC cells. (A) Stable overexpression and suppression of CEACAM6 in GC cells. (B) CEACAM6 expression was analyzed by immunofluorescence, and more CEACAM6 expression was detected in CEACAM6-overexpressing cells than that in control cells (200×). Blue: DAPI; red: CEACAM6. (C) Overexpression of CEACAM6 in SGC-7901 and MKN-45 cells induced a mesenchymal morphology, whereas knockdown of CEACAM6 in MKN-28 cells induced an epithelial morphology (200×). (D) Immunostaining of F-actin in CEACAM6-overexpressing cells and control cells (400×). Red: F-actin; blue: DAPI.

Our previous results implied that MKN-28 GC cells inherently express high levels of CEACAM6, whereas SGC-7901 and MKN-45 GC cells express low levels [12]. Overexpression of CEACAM6 in SGC-7901 and MKN-45 GC cells and attenuation of CEACAM6 expression in MKN-28 GC cells by pL/shRNA/shR-CEACAM6 lentiviral vectors were confirmed at the protein level by western blot analysis (Fig. 1A). Immunofluorescence assays always showed that SGC-7901-CEACAM6 cells expressed more CEACAM6 protein than SGC-7901-NC cells (Fig. 1B).

Interestingly, cell morphology was altered following CEACAM6 attenuation and overexpression. Compared with the control cells, SGC-7901-CEACAM6 and MKN-45-CEACAM6 cells exhibited a more mesenchymal-like morphology, and were spindle- and fusiform-shaped (Fig. 1C). Conversely, typical transition from mesenchymal to epithelial morphology was detected in MKN-28-sh cells compared with MKN-28-nc cells (Fig. 1C). However, it was unclear if CEACAM6 overexpression had an effect on the cytoskeleton, thus immunofluorescent staining of F-actin staining was performed. Interestingly, larger-sized fusiform-shaped cells were observed for MKN-45-CEACAM6 and SGC-7901-CEACAM6 lines compared with the control cells (Fig. 1D).

### CEACAM6 is negatively associated with E-cadherin in GC tissues

**Figure 2:**
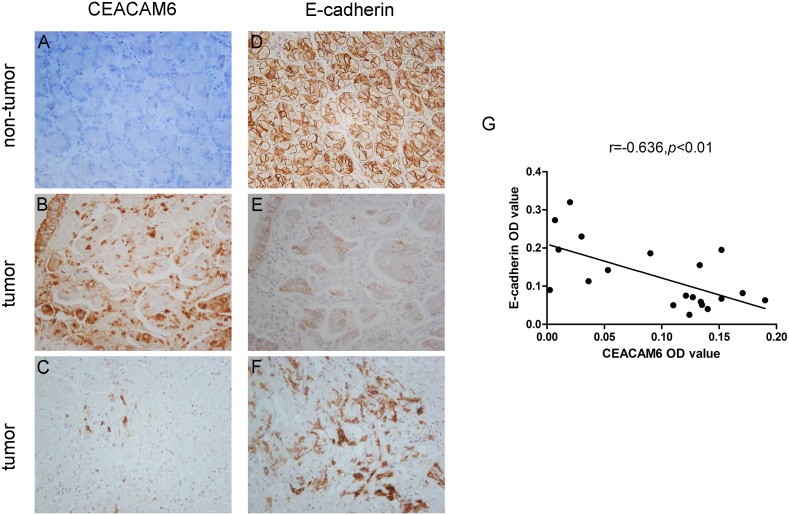
Expression of CEACAM6 and E-cadherin in gastric tissues. (A) Negative CEACAM6 expression in non-tumor gastric mucosa. (B, C) Positive or negative CEACAM6 expression in GC samples. (D) Strong positive E-cadherin expression in non-tumorous gastric mucosa. (E, F) Negative or positive E-cadherin expression in GC samples. (G) Negative correlation between CEACAM6 and E-cadherin expression was detected in GC tissues (R = −0.636, *P*<0.01) (200×).

Immunohistochemical analysis was used to determine the levels of E-cadherin expression in cancer tissues and paired adjacent non-tumorous tissues. Immunohistochemical staining showed E-cadherin expression was lower in tumor tissues than that in adjacent non-tumorous tissues, and revealed that E-cadherin is mainly located in the cytomembrane of epithelial cells (Fig. 2D, E, F). Next, we investigated the relationship between E-cadherin expression and clinicopathological features of GC, and found that downregulated E-cadherin was associated with depth of invasion (*P* = 0.046), lymph node metastasis (*P* = 0.013), and TNM stage (*P* = 0.035), but not with other clinicopathological factors including sex, age, or differentiation (Table 1). Additionally, CEACAM6 was negatively correlated with EMT in pancreatic carcinomas [19] and CEACAM6 suppression could increase E-cadherin promoter activity in colorectal cancer [20].

**Table 1:** Association between E-cadherin expression and clinicopathological factors of gastric cancer patients.

Clinicopathologic	Number(n = 93)	E-cadherin		*P*
Parameters		Negative(n = 53)	Positive(n = 40)	
Age(years)				
<65	66	39	27	0.522
≥65	27	14	13	
Gender				
Male	69	40	29	0.746
Female	24	13	11	
Differentiation				
Well to moderate	31	19	12	0.554
Poor	62	34	28	
T stage				
T1, T2	23	9	14	0.046
T3, T4	70	44	26	
Tumor location				
Gastric fundus	10	4	6	
Gastric corpus	23	13	10	0.496
Pylorus	60	36	24	
Tumor size				
≤5 cm	57	33	24	0.824
>5 cm	36	20	16	
Lymph node metastasis				
Yes	64	42	22	0.013
No	29	11	18	
TNM stage				
I, II	33	14	19	0.035
III, IV	60	39	21	

Furthermore, we investigated the correlation between E-cadherin and CEACAM6 expression in GCs by serial section immunohistochemistry (20 samples). CEACAM6 expression in tumor tissues was higher than that in matched non-tumorous tissues (Fig. 2A, B, C), consistent with our previous results [12]. Interestingly, we found that CEACAM6 expression was negatively correlated with E-cadherin expression in GC tissues by Pearson correlation coefficient analysis (*P*<0.01; Fig. 2G).

### CEACAM6 induces EMT in GC cells

**Figure 3:**
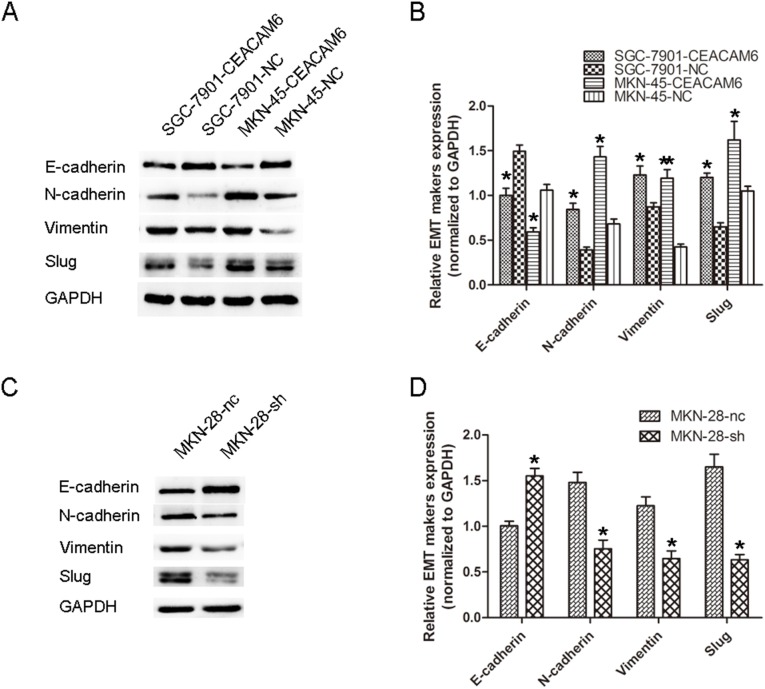
CEACAM6 induces EMT in GC. (A, C) The protein levels of EMT markers were assayed by western blot analysis in GC cells with CEACAM6 overexpression or knockdown. (B, D) Relative EMT marker expression in CEACAM6-overexpressing and -silenced GC cells were calculated by gray scale ratio. The results are means of three independent experiments in the bar graphs. *P<0.05, **P<0.01.

EMT-related proteins in GC cells were evaluated by western blot analysis. We observed that the N-cadherin, Vimentin and Slug protein levels were increased, while E-cadherin was decreased in CEACAM6-overexpressing cells compared with their respective control cells (Fig. 3A, B). Converse results were obtained after CEACAM6 was knocked down in MKN-28 cells (Fig. 3C, D). These results suggested a functional role for CEACAM6 in regulating EMT in GC cells. Moreover, these observations in GC cells were similar with the findings in GC tissue.

### CEACAM6 elevates the activity of MMP-9 in GC cells

**Figure 4:**
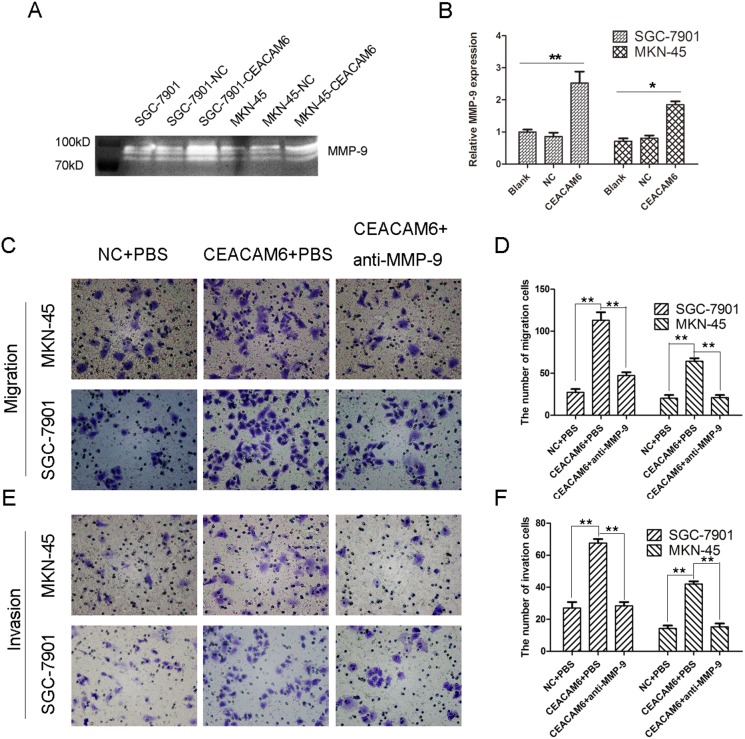
Effect of CEACAM6 on MMP-9 activity, and cell migratory and invasive ability in GC cells. (A) The supernatant of GC cells was collected after 24 h. Then the MMP-9 activity was examined by gelatin zymography. (B) Relative MMP-9 expression in GC cells. (C, E) Transwell assay. Images show cells that had traveled through the micropore membrane with or without matrigel (200×). (D, F) Histograms showed the numbers of migrating cells and invading cells. The results are means of three independent experiments in the bar graphs. *P<0.05, **P<0.01.

It is well known that adhesion, degradation and migration are major issues in cancer metastasis. Gelatin zymography was carried out to examine the effects of CEACAM6 on the activity of MMP-9, which is important in ECM degradation. MMP-9 activity in SGC-7901-CEACAM6 and MKN-45-CEACAM6 cells was increased compared with their control groups (Fig. 4A, B). We next investigated whether the increased activity of MMP-9 induced by CEACAM6 in GC cells resulted in a true increment in the number of invading and migrating cells. We thus examined the contribution of active anti-MMP-9 antibody or PBS to CEACAM6-overexpressing GC cells. As expected, there were significant differences in the number of invading and migrating cells in antibody-treated cells compared with PBS-treated cells (*P*<0.01; Fig. 4C, D, E, F).

### CEACAM6 upregulates phosphorylated AKT in GC cells

**Figure 5:**
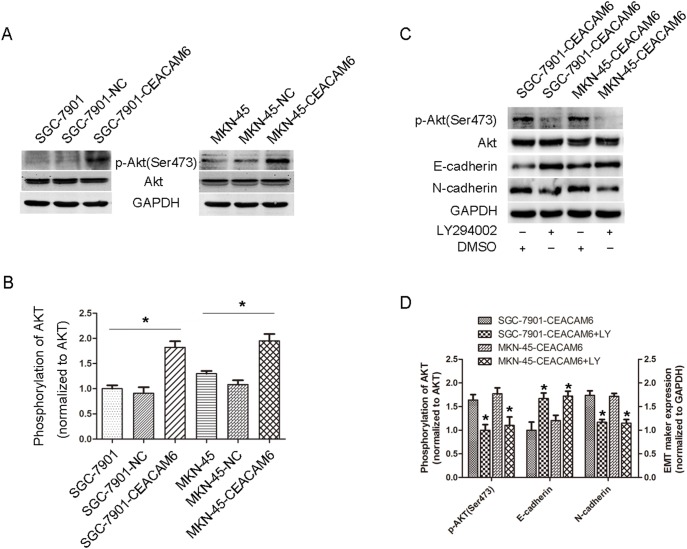
Effect of CEACAM6 on AKT activity in GC cells. (A) Western blot analysis showed that overexpression of CEACAM6 increased p-AKT (Ser473) phosphorylation. GAPDH and total AKT were used as loading controls. (B) The quantification of p-AKT (Ser473) expression in GC cells. (C) E-cadherin expression was increased while N-cadherin expression was decreased in CEACAM6-overexpressing GC cells treated with LY294002, a PI3K inhibitor. (D) Relative E-cadherin and N-cadherin expression in CEACAM6-overexpressing and LY294002-treated GC cells were calculated by gray scale ratio. The results are means of three independent experiments in the bar graphs. *P<0.05.

We previously observed that SRC activity was induced by CEACAM6 in SGC-7901 GC cells. AKT is a downstream protein of SRC and is dramatically associated with EMT, invasion and metastasis in tumor progression. We therefore evaluated AKT activity in CEACAM6-overexpressing GC cells. Western blotting showed that AKT phosphorylation at serine 473 was markedly increased in CEACAM6-overexpressing GC cells compared with the control groups (Fig. 5A, B). To examine whether EMT changes were induced by CEACAM6 via the PI3K/AKT pathway, SGC-7901-CEACAM6 and MKN-45-CEACAM6 cells were treated with 100 nM LY294002 for 24 h. Interestingly, E-cadherin was increased and N-cadherin was reduced in LY294002-treated CEACAM6-overexpressing cells compared with the controls, as measured by western blot analysis (Fig. 5C, D).

### CEACAM6 promotes peritoneal spreading in vivo

**Figure 6:**
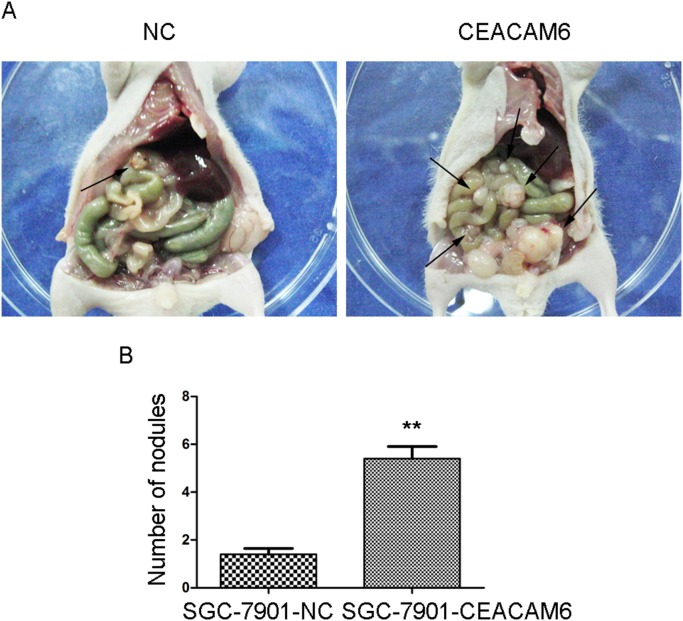
Effect of CEACAM6 overexpression on peritoneal spread and metastasis. (A) Increased numbers of metastatic nodules were observed in the SGC-7901-CEACAM6 group than the control group, as indicated by the black arrows. (B) Average number of peritoneal nodules in two groups. More nodules occurred in the SGC-7901-CEACAM6 group than the SGC-7901-NC group.

Finally, we examined the effects of CEACAM6 overexpression on peritoneal spreading and metastasis *in* *vivo*. We injected SGC-7901-CEACAM6 and SGC-7901-NC cells into the abdomens of nude mice. Extensive peritoneal spread was observed in the SGC-7901-CEACAM6 group compared with the SGC-7901-NC group (Fig. 6A). There were significantly more visible peritoneal nodules in the SGC-7901-CEACAM6 group than in the control group (5.400±0.510 *vs.* 1.400±0.245, *P*<0.01; Fig. 6B). Additionally, overexpression of CEACAM6 enhancing metastasis *in* *vivo* has also been reported in pancreatic cancer [19].

## Discussion

GC is the second cause of cancer-related death worldwide [1]. Increasing evidence indicates that CEACAM6 plays an important role in gastrointestinal cancer progression [7], [20]–[22], and CEACAM6 is more widely distributed than CEA (CEACAM5) in normal tissues, with significant expression in many epithelia [4]. CEACAM6 influences intracellular signaling events through homophilic and heterophilic adhesion with other CEACAMs or integrins [23], [24]. The purpose of this study was to examine the contributions of GPI-anchored protein CEACAM6 in GC progression.

Interestingly, we observed that cell morphology and cytoskeletal structure was altered by stable upregulation and downregulation CEACAM6 in GC cells. CEACAM6-overexpressing cells were more mesenchymal-like and exhibited a spindle and fusiform shape, and more actin stress fibers were detected compared with the control groups, in a process defined as EMT. The mesenchymal phenotype endows cells with more migratory and invasive properties [15]. This observation was consistent with the *in* *vivo* results whereby CEACAM6 induced extensive peritoneal spreading in nude mice. MET was observed following silencing of CEACAM6 in GC cells. These observations demonstrated that CEACAM6 may be involved in inducing EMT in GC. We next examined the correlation between CEACAM6 and E-cadherin, a marker of EMT, in GC tissues by immunohistochemical staining. As expected, significant negative correlation was observed between CEACAM6 and E-cadherin, and E-cadherin expression was associated with depth of tumor invasion, lymph node metastasis and TNM stage in GC tissues. Additionally, CEACAM6 promoted lymph node metastasis (*P* = 0.001) in 98 GC tissues in our previous study [12]. Previous results showed that CEACAM6 attenuation increased E-cadherin promoter activity in colorectal cancer [20]. Based on the abovementioned findings, we postulated that CEACAM6 promoted GC invasion and metastasis by inducing EMT and may serve as a mesenchymal marker.

Metastasis is the leading cause of cancer-associated death but has been difficult to study because it involves a series of rare, stochastic events. A number of potential signaling pathways relevant to cancer metastasis have been illustrated, including the PI3K/AKT, MAPK/ERK, FAK-SRC, JAK/STAT, NF-kβ, and MMPs pathways [25]–[27]. In this study, overexpression of CEACAM6 elevated MMP-9 activity, and anti-MMP-9 antibody could reverse the increasing invasive and migratory properties induced by CEACAM6. EMT initiation is significantly correlated with cancer metastasis, and induces stem cell properties, prevents apoptosis and senescence, and contributes to immunosuppression in cancer progression [15]. EMT can be induced through activation of the PI3K/AKT signaling pathway [28], [29]. CEACAM6 functions as a intercellular adhesion molecule and can form bigger lipid rafts by interacting with itself or other CEACAMs, thus activating the downstream signaling cascades, such as the integrin and PI3K/AKT pathways [10], [30]. Therefore we examined the effects of CEACAM6 overexpression on the levels of AKT phosphorylation in GC cells. Phosphorylated AKT levels were increased in CEACAM6-overexpressing cells, consistent with previous results noted in pancreatic carcinomas [8], [11]. According to these observations, we propose a hypothesis whereby CEACAM6-induced EMT occurs through activation of the PI3K/AKT signaling cascade in GC. Moreover, CEACAM6-overexpressing cells treated with LY294002, a PI3K inhibitor, underwent a reversal of EMT via MET. Thus, we conclude that CEACAM6 induces EMT by activating the PI3K/AKT signaling pathway in GC and may serve as a potential target in tumor treatment.

In conclusion, we have illustrated that CEACAM6 acts as an oncogene by promoting EMT via PI3K/AKT activation in GC. Furthermore, E-cadherin is markedly downregulated in GC tissues than paired adjacent non-tumorous tissues. CEACAM6 promotes invasion and metastasis *in* *vitro* and *in* *vivo*, and may be a potential new diagnostic and prognostic marker and novel target for GC therapy.

## Supporting Information

Checklist S1 **The ARRIVE guidelines.** All in vivo experiments in our manuscript were exported in accordance with the ARRIVE guidelines.
(DOC)
